# Effect of fructo-oligosaccharides on growth performance and meat quality in broilers

**DOI:** 10.3389/fvets.2024.1485077

**Published:** 2025-01-07

**Authors:** ZhiHui Yan, XiaoWu Tang, RunTao Wu, Can Yang, YunMiao Jiang, Xuan Wang, QingHai Tang, YongLing Hu, LeLi Wang, Zhi Jiang

**Affiliations:** ^1^College of Life Sciences, Hunan Provincial Key Laboratory of Biological Resources Protection and Utilization in Nanyue Mountain Area, Hengyang Normal University, Hengyang, Hunan, China; ^2^College of Bioengineering, Hunan Vocational Technical College of Environment and Biology, Hengyang, Hunan, China; ^3^Key Laboratory of Agro-ecological Processes in Subtropical Region, Institute of Subtropical Agriculture, Chinese Academy of Sciences, Changsha, China; ^4^YiMin Ecological Agriculture Development Co., Ltd., Hengyang, China

**Keywords:** fructo-oligosaccharides, growth performance, texture characteristics, myofibrillar morphology, muscle

## Abstract

This study investigated the fructo-oligosaccharides (FOS) on growth performance and meat quality in broilers. Total 160 Xianghuang broilers aged 2 months were randomly assigned into 2 groups, CON (control), FOS (supplemented 0.5% fructo-oligosaccharides in diet). After 38 days, the breast, thigh muscle and liver samples were collected for further analysis. Results showed that no significant effect of 0.5% FOS on growth performance such as average daily gain (ADG), average daily feed intake (ADFI) or feed-to-gain ratio (F:G) were observed (*P* > 0.05). Broilers in FOS group had a yellower breast than that in CON group (*P* < 0.05). Breast pH_45min_ and thigh pH_24h_ value of FOS group were greater than that in CON group (*P* < 0.05). Max shear force and work of shear of cooked breast (*pectoralis major*) muscle was lower in FOS group compared with CON group (*P* < 0.05). Hardness (*P* = 0.065), fracturability (*P* = 0.063), gumminess (*P* = 0.079), chewiness (*P* = 0.080) of cooked thigh meat tended to be higher in FOS group compared to the CON group. Addition of 0.5% FOS resulted in lower thigh total superoxide dismutase (T-SOD) activity compared to CON group (*P* < 0.05). The malonaldehyde (MDA) concentration (*P* = 0.066) of breast muscle tended to be lower in FOS group compared with CON group. There was an increasing trend for total antioxidant capacity (T-AOC) activity of thigh muscle in FOS group compared to CON group (*P* = 0.053). Relative mRNA expression of breast catalase (CAT), superoxide dismutase 1 (SOD1), thioredoxin reductase 1 (TXNRD) were up-regulated by FOS supplementation compared with CON group (*P* < 0.05). In conclusion, FOS can be utilized at 0.5 % to improve meat quality such as elevating pH value, yellowness and decreasing max shear force of muscle through enhancing the antioxidant activity in broilers.

## 1 Introduction

Fructo-oligosaccharide is water-soluble dietary fiber which formed by D-fructose and sucrose binding by *β*-1,2 glycosidic bonds (1). It exists in wheat, potatoes, onion, garlic, bananas and other plants. FOS was reported as involved in the fat metabolism through mobilizing the intestinal bacteria and their metabolites. Supplementation of 1 g FOS per liter of water increased the mRNA expression of genes related to fat digestion and absorption, leucine and isoleucine biosynthesis in ileal mucosa of Taiping chickens (2). Supplemented with 5 g/kg FOS significantly inhibited cecal *E.coli* growth in 3-and 5-wk-old broilers (3), increased microbial diversity of ileal mucosa in 21-day-old broilers when compared with wheat-corn-soybean meal based diet (4). Cecal abundance of *Escherichia coli* decreased but *Bifidobacterium* spp. and *Lactobacillus* spp. increased after supplementation of FOS and beneficial microorganisms (*Bifidobacterium animalis, Enterococcus faecium, Lactobacillus reuteri, Pediococcus acidilactici*) in heat-stressed broilers (5). Increasing colonization of *B. subtills* in broilers’ gastrointestinal tract would be beneficial to their musculoskeletal health (6). Visual appearance of broilers’ thigh muscles was improved by *Lactobacillus* through increasing xanthophyll accumulation in soft tissues (7). *B. subtilis*-fed broilers had greater water holding capacity, better taste (flavor, texture, preference, and general aspect) in leg muscle, and these probiotic effects were greater in 0.5 g/kg group than in the 0.25 g/kg group (6). Further, broilers muscle is rich in polyunsaturated fatty acids (8), which makes it sensitive to oxidative deterioration. Due to the effect on bacterial fermentation in the intestine, mineral absorption increased when broilers supplemented with 0.4% (9) or 0.5% (10) FOS (11). Supplemented with coated trace minerals (Cu, Fe, Mn, Zn, Se) in broilers’ diet could decrease both serum and muscle MDA levels and then reduce drip loss of meat (12). Mineral element Zn and Cu is essential for SOD activity. Antioxidant enzymes such as SOD and glutathione peroxidase are able to protect polyunsaturated fatty acids in chicken muscle from free radicals and reactive oxygen species damage. Whether meat quality even meat texture could be improved by this 0.5% relatively high dosage FOS supplementation in broilers is still not well known.

There was positively correlation between the ratio of type I myofiber and antioxidized activities, pH value postmortem, intramuscular fat and saturated fatty acid (SFAs) content in Yak beef *Semitendinosus* muscles (13). Type IIB myofiber was fast glycolytic myofiber, it contained two-thirds of myoglobin as type I fibers (14), leading to a paler meta color. Compared to glycolytic-type fiber (Type IIX and IIB), oxidative-type muscle fibers (Type I and IIA) had smaller diameters and higher density (15), which contributes to decrease in shear force and increase in meta tenderness (16). Xianghuang broiler is a slower growing breed. Results showed that the breast (*pectoralis major*, PM) muscle only made up of type IIB fibers in slow-growing Xueshan chicken and fast-growing Ross 308 broiler (17) or Japanese quail (18) but little type I fibers could be found in thigh (*gastrocnemius*, GAS) muscle of Xueshan and Ross 308 broilers (17). If breast muscle and thigh muscle of Xianghuang broilers respond different to this relatively high dosage of FOS still need to further study. Therefore, we performed a comparative analysis of the effect of 0.5% of dietary FOS on breast and thigh muscle. The objectives of the current work were to evaluate the effect of dietary FOS on growth performance and meat quality in Xianghuang broilers. We hypothesized that high dosage FOS supplementation would improve meat quality through affecting muscle metabolic and antioxidant function in broilers.

## 2 Materials and methods

### 2.1 Animal ethic statement

Animal work was approved by the Animal Care and Use Committee of Hengyang Normal University, protocol HNUACUC-B202201005.

### 2.2 Animals and experimental treatments

A total of 160 male Xianghuang broilers (0.876 ± 0.149 kg, 2 months old) were randomly assigned to 2 treatments. Each treatment had 8 replicates with 10 broilers per replicate cage. Broilers were fed a corn-soybean meal-based diet (Table 1) that met the nutritional recommendations for yellow-feathered broilers (19), but with or without 0.5% fructo-oligosaccharides, and named as FOS or CON, respectively. FOS was kindly provided by Shandong Longli Biological Technology Co., Ltd. (Shandong, China). Broilers were raised in floor commercial pens (about 0.1 m^2^/bird) with free access to semi-powder semi-pellet feed and water over the total period of 38 days. Room temperatures were maintained at 22°C by indoor air conditioning. Light was provided for 16 h at 10 lux throughout the experimental period.

**Table 1:** Calculated ingredient composition of Xianghuang broilers’ diets (%, as-fed basis).

Formular		Nutrient levels^1^	
Name	Content	Name	Content
Ingredients		ME, kcal/kg	2,800
Corn	69.42	CP	15.50
Soybean meal (43% CP)	22.37	Ca	0.90
Wheat bran	2.96	P	0.60
Limestone	1.39	Digestible P	0.41
CaHPO_4_	1.55	NaCl	0.30
Vitamin premix^2^	1.00	Lys	0.73
Mineral premix^2^	1.00	Met+Cys	0.55
NaCl	0.27	Thr	0.64
Met	0.04	Trp	0.20
Total	100.00		

1 Nutrient levels were all calculated values, and amino acids were standardized ileal digestible amino acids.

2 Provided the following quantities of vitamins and micro-minerals per kilogram of complete diet: vitamin A as retinyl acetate, 4,000 IU; vitamin D_3_ as cholecalciferol, 800 IU; vitamin E as DL-alpha tocopheryl acetate, 8 IU; vitamin K as menadione dimethylpyrimidinol bisulfite, 0.5 mg; thiamin as thiamine mononitrate, 1.0 mg; riboflavin, 1.8 mg; pyridoxine as pyridoxine hydrochloride, 3.0 mg; vitamin B_12_, 3.0 μg; D-pantothenic acid as D-calcium pantothenate, 10.0 mg; niacin, 11.0 mg; folic acid, 0.25 mg; biotin, 0.1 mg; choline chloride 900 mg; Cu, 8 mg as copper sulfate; Fe, 80 mg as ferrous sulfate; I, 0.35 mg as ethylenediamine dihydride; Mn, 60 mg as manganese sulfate; Se, 0.3 mg as sodium selenite; and Zn, 60 mg as zinc sulfate.

### 2.3 Sample collection

All birds were weighed every week per replicate cage. Feed intake/leftover was recorded every day. Body weight gain and feed conversion ratio were calculated. On d 38, all birds were weighed individually and 2 medium-weight birds per cage were randomly taken and euthanized by carbon dioxide and then cervical dislocation. Liver, boned right breast and thigh muscle were weighed and their percentage were calculated as hot tissue weight/live body weight × 100%. After weighing the eviscerated carcasses, the giblets were removed and the head and toes of the chicken were preserved. Left breast and thigh muscles of 5 cm length were removed along the breastbone and placed in 4% paraformaldehyde for histological analysis. Residual muscle and liver were collected and stored frozen (−80°C) until gene analyses and enzymes analyses.

### 2.4 Meat quality and nutrient measurements

Meat color such as lightness (L*), redness (a*), yellowness (b*) were determined at 45 min and 24 h postmortem on left 3 cm thick deboned muscle sample using colorimeter (CR-410, Kinica Minolta Sensing Inc., Osaka, Japan). The evaluation was carried out three times on the posterior surface of the skinless breast and thigh muscle. The pH measurement was taken from three different regions of each muscle with portable pH probe (Matthaus pH Star, Germany). Drip loss of muscle was measured as follows, approximately 2 g of left fillet was weighed and suspended on a barbless hook in an inverted plastic cup, suspended for 24 h at 4°C before being removed from the hook, and reweighed. Approximately 5 g of right muscle was weighed, cooked on a steamer, boiling water (95°C) vapor in the bottom of the steamer rise and through the pore to boil the meat for 30 min until the inner temperature reached to 70°C, they were reweighed after these cooked samples cooled to room temperature, and cooking loss was expressed as percentage loss during cooking. Cooked samples were placed in silver paper and held at −20°C until texture profile analysis (TPA) and shear force analysis. Muscles and liver were freeze-dried for 72 h (YAMATO DC801, Japan). Crude fat content was extracted by petroleum ether under Soxhlet extraction method (20). Crude protein content were determined by Kjeldahl method (20).

### 2.5 Myofibrillar morphology

Muscle samples from the 4% polyformaldehyde were washed in running water overnight, treated with increasing concentrations of ethanol, transparence with xylene and embedded in solid paraffin. Slides of 5 μm thick were obtained on rotary microtome (Leica RM2135, Leica Microsystems, Wetzlar, Germany), and then hematoxylin and eosin staining. Images were recorded by Leica inverted microscope (Leica DM500) with camera (Leica MC170 HD). Fiber diameter, cross-sectional area and density were analyzed from 80 fibers per broiler using Image-Pro Plus software (Media Cybernetics Inc., Silver Spring, MD).

### 2.6 Shear force and texture parameters

Raw and cooked breast and thigh muscle were cut into 1.5 cm × 1.5 cm × 0.5 cm (height) parallel to the muscle fiber orientation 1 day postmortem. Shear force of muscle or meat were measured using Warner-Bratzler HDP/BSW under toughness program fitted with a 50-kg load cell on Texture Analyzer (TA. XT. Plus. Stable Micro systems, United Kingdom). Test settings included a button type trigger, 62 mm travel distance, 2 mm/s test speed, and 10 mm/s post-test speed (21). Max shear force (kg) and total shear energy (work of shear, kg.sec) were recorded.

Texture profile analysis (TPA) of muscle and meat was measured on Texture Analyzer (TA. XT. Plus. Stable Micro systems, United Kingdom) using probe P36R under TPA program. Testing conditions were as follows, holding time was 2 s, trigger force was 0.1 g, test speed was 5.0 mm/s (pre-test), 1 mm/s (test), and 5.0 mm/s (post-test) to reach a 50% compression (22). TPA parameters including hardness, fracturability, adhesiveness, springiness, cohesiveness, gumminess, chewiness, resilience were calculated from the Texture Expert version 1.0 software. Measurements were performed in triplicate for each meat sample and the average value was used for statistical analysis.

### 2.7 Antioxidant status measurement

Approximately 0.5 g fresh muscle or liver were homogenized in 4.5 mL of 0.9% NaCl solution using tissue grinder (SCIENTZ-12, Xinzhi Biotech logy, Ningbo, China), and then centrifuged (2,500 r/min (1845 g), 15 min, 4°C) to collect supernatant. Activities of total antioxidant capacity (T-AOC), superoxide dismutase (SOD), catalase (CAT), glutathione peroxidase (GSHPX) and MDA concentration were tested according method mentioned in Tan et al. (23). Briefly, activity of T-AOC (mmol/L) was analyzed using its OD 593 nm value compared with standard curve of FeSO_4_. The Unit of CAT activity was defined as mg of hydrolyzed H_2_O_2_ in 1 min per mg protein of sample. One Unit of SOD enzyme was defined as the amount of enzyme that inhibits 50% of lighting reaction of nitroblue tetrazolium. Supernatant was extracted in 10% trichloroacetic acid and then was used to test MDA concentration. Protein concentrations were determined using Bradford method with bovine albumin as the standard.

### 2.8 Gene expression analysis

Total RNA from muscle and liver was extracted using Trizol reagent (Takara, Dalian, China). Sample concentration and quality were determined on BioSpec-nano (Shimadzu, Japan). 1.0 μg of total RNA was reverse-transcribed into cDNA using the Reverse Transcription Reagent Kit (Aikerui, Changsha, China). The mRNA expression levels of genes (Table 2) were determined using Real-time PCR performed on an QuantStudio 3 (Applied Biosystems, Branchburg, NJ) using SYBR Green quantitative PCR mix (Aikerui, Changsha, China). The 2^−△△Ct^ method (24) was used to calculate the gene expression relative to *β*-actin which was used as housekeeping gene.

**Table 2:** Sequences of primers used for quantitative real-time PCR.

Name^1^	Sequence (5–3′)	Product length	NCBI reference sequence
β-actin	F: CATTGTCCACCGCAAATGCT	108	NM_205518. 1
	R: AGCCATGCCAATCTCGTCTT		
HMOX1	F: ACACCCGCTATTTGGGAGAC	167	NM_205344.1
	R: AAGGGCATTCATTCGGGACC		
NFE2L2	F: ATGTCACCCTGCCCTTAGAG	189	NM_205117.1
	R: TGCAGAAGAGGTGATGACGG		
CAT	F: GCCACATGGTGACTACCCTC	107	NM_001031215.2
	R: TGTTGCTAGGGTCATACGCC		
SOD1	F: CACGGTGGACCAAAAGATGC	123	NM_205064.1
	R: GATGCAGTGTGGTCCGGTAA		
NQO1	F: GAGCGAAGTTCAGCCCAGTAT	151	NM_001277619.1
	R: CATGGCGTGGTTGAAAGAGG		
TXNRD1	F: ATCGCTATGGCTGACCTGTG	136	NM_001030762.3
	R: GGTGGCTAACTCCCCTCTTG		
IL1β	F: TGCCTGCAGAAGAAGCCTCG	204	NM_204524.1
	R: GACGGGCTCAAAAACCTCCT		
IL8L2	F: CCTAACCATGAACGGCAAGC	174	NM_205498.1
	R: CTTGGCGTCAGCTTCACATC		
TNFα	F: GGGACGGCCTTTACTTCGTA	113	MF000729.1
	R: GTCTTTGGGGTACTCCTCGG		

1 HMOX1, heme oxygenase 1; NFE2L, nuclear factor, erythroid 2 like 2; CAT, catalase; SOD1, superoxide dismutase 1; NQO1, NAD(P)H quinone dehydrogenase 1; TXNRD, thioredoxin reductase 1; IL1β, interleukin 1, beta; IL8L2, interleukin 8-like 2; TNFa, tumor necrosis factor alpha.

### 2.9 Statistical analysis

Pen was considered as the experimental unit. All experimental data were analyzed by One-way ANOVA procedure of SAS 8.2 software package (SAS Inst. Inc., Cary, NC). Differences between the means were determined with t tests. Data were presented as mean ± standard error. A value of *P* < 0.05 was considered significant and 0.05 < *P* < 0.10 was reported as a trend.

## 3 Results

### 3.1 Growth performance

Growth performance such as ADFI, ADG or F:G was not affected by dietary FOS treatment (*P* > 0.05) (Table 3).

**Table 3:** Effect of fructo-oligosaccharides on growth performance of Xianghuang broilers^1^.

Items^1^	Control	FOS	*P*-value
Initial BW, day 85, kg	0.85 ± 0.03	0.90 ± 0.13	0.495
Final BW, day 123, kg	1.20 ± 0.04	1.34 ± 0.13	0.084
First 10 days			
ADFI, g/d	69.75 ± 4.50	66.38 ± 2.25	0.228
ADG, g/d	8.69 ± 1.39	9.13 ± 2.32	0.758
F:G, g/g	8.15 ± 1.07	7.65 ± 2.01	0.682
Week 2			
ADFI, g/d	69.78 ± 5.15	64.64 ± 6.74	0.271
ADG, g/d	9.11 ± 3.12	10.36 ± 1.96	0.523
F:G, g/g	8.52 ± 3.37	6.33 ± 0.73	0.252
Week 3			
ADFI, g/d	82.66 ± 3.83	82.29 ± 4.87	0.908
ADG, g/d	8.48 ± 4.78	5.63 ± 1.73	0.304
F:G, g/g	12.03 ± 5.74	15.51 ± 3.84	0.352
Week 4			
ADFI, g/d	79.8 ± 6.05	77.13 ± 9.19	0.645
ADG, g/d	11.88 ± 1.38	11.16 ± 2.53	0.638
F:G, g/g	6.82 ± 1.19	7.17 ± 1.66	0.740
Week 5			
ADFI, g/d	80.07 ± 14.34	89.07 ± 9.04	0.329
ADG, g/d	8.04 ± 3.00	22.5 ± 20.59	0.214
F:G, g/g	7.24 ± 2.71	6.78 ± 1.72	0.783
Total 38 days			
ADFI, g/d	75.89 ± 3.51	75.15 ± 1.56	0.714
ADG, g/d	9.19 ± 0.42	11.55 ± 3.81	0.266
F:G, g/g	8.26 ± 0.30	8.44 ± 1.5	0.817

1 BW, body weight; ADG, average daily gain; ADFI, average daily feed intake; F:G, ratio of feed to gain. FOS 0.5%.

### 3.2 Carcass traits

Dietary FOS supplementation did not affect breast or thigh muscle yield, liver weight and percentage, eviscerated carcass yield of Xianghuang broilers (*P* > 0.05) (Table 4).

**Table 4:** Effect of fructo-oligosaccharides on carcass parameters in Xianghuang broilers.

Items	Control	FOS	*P*-value
Weight, g			
Body weight, Kg	1.15 ± 0.18	1.16 ± 0.09	0.886
Right breast muscle	71.89 ± 13.68	67.95 ± 7.93	0.523
Right thigh muscle	77.82 ± 15.90	78.94 ± 15.16	0.895
Liver	19.64 ± 5.58	17.08 ± 2.90	0.302
Full net chamber^1^	882.56 ± 119.08	849.75 ± 104.94	0.594
Ratio to body weight, %			
Right breast muscle	6.23 ± 0.44	5.87 ± 0.79	0.315
Right thigh muscle	6.73 ± 0.42	6.79 ± 1.18	0.892
Liver	1.69 ± 0.25	1.47 ± 0.21	0.100
Eviscerated carcass yield	77.3 ± 8.66	73.15 ± 7.76	0.365

1 Head and chicken toe were preserved when eviscerated carcass were weighed.

### 3.3 Meat quality

Breast filets from FOS birds had higher b^*^_24h_ value than that in CON group (*P* < 0.05) (Table 5). The pH_45min_ value of breast fillet and pH_24h_ of thigh fillet were significantly higher for FOS broilers when compared to CON broilers (*P* < 0.05). For breast muscle, no significant difference was observed on drip loss, cooking loss, pH_24h_, meat color (L*, a*, b*) at 45 min, L* and a* index at 24 h between treatments (*P* > 0.05). There was no significant difference in drip loss, pH_45min_ value, meat color at 45 min and 24 h of thigh muscle (*P* > 0.05). Cooking loss of thigh fillet tended to be affected by diet (*P* = 0.071), with decreased value occurring in FOS birds compared to CON group. Crude protein and fat content of breast and thigh muscle or liver were not affected by FOS supplementation (*P* > 0.05) expect that FOS group had lower crude protein content in liver compared to CON group (*P* < 0.05).

**Table 5:** Effect of fructo-oligosaccharides on meat quality in Xianghuang broilers.

Items	Control	FOS	*P*-value
Breast muscle			
Drip loss, %	1.93 ± 0.54	1.78 ± 0.53	0.613
Cooking loss, %	32.13 ± 2.36	32.18 ± 1.27	0.957
pH_45 min_	6.21 ± 0.21^B^	6.48 ± 0.24^A^	0.041
pH_24 h_	5.74 ± 0.09	5.78 ± 0.10	0.481
Lightness (L^*^)_45 min_	53.01 ± 4.2	53.87 ± 1.33	0.615
Redness (a^*^)_45 min_	2.92 ± 1.91	2.78 ± 0.89	0.863
Yellowness (b^*^)_45 min_	9.44 ± 1.99	10.84 ± 1.21	0.137
L^*^_24 h_	60.92 ± 3.28	59.00 ± 3.60	0.317
a^*^_24 h_	1.86 ± 1.48	2.1 ± 0.86	0.712
b^*^_24 h_	10.24 ± 2.04^B^	13.15 ± 2.30^A^	0.032
Crude fat^1^, %	2.25 ± 0.78	3.13 ± 1.17	0.126
Crude protein^1^, %	22.41 ± 2.09	22.96 ± 2.45	0.661
Thigh muscle			
Drip loss, %	1.82 ± 0.59	2.11 ± 0.73	0.432
Cooking loss, %	42.7 ± 5.73	35.85 ± 5.97	0.071
pH_45 min_	6.55 ± 0.24	6.58 ± 0.11	0.755
pH_24 h_	6.02 ± 0.17^B^	6.22 ± 0.12^A^	0.003
L^*^_45 min_	51.71 ± 1.4	50.4 ± 2.76	0.286
a^*^_45 min_	5.12 ± 1.48	5.63 ± 0.78	0.430
b^*^_45 min_	7.86 ± 1.22	7.79 ± 3.80	0.962
L^*^_24 h_	54.58 ± 6.48	50.72 ± 3.3	0.186
a^*^_24 h_	5.98 ± 1.92	6.88 ± 4.32	0.625
b^*^_24 h_	10.29 ± 6.07	9.94 ± 2.94	0.893
Crude fat^1^, %	2.44 ± 1.08	2.61 ± 0.72	0.729
Crude protein^1^, %	21.49 ± 3.74	19.83 ± 4.32	0.458
Liver			
Crude fat^1^, %	3.90 ± 0.58	3.16 ± 1.16	0.155
Crude protein^1^, %	28.65 ± 2.35^A^	25.06 ± 1.19^B^	0.004

1 Crude protein and crude fat concentrations were given by fresh muscle or liver sample. Values in a row without common superscripts differ significantly at *P* < 0.05.

### 3.4 Myofibrillar morphology

**Figure 1:**
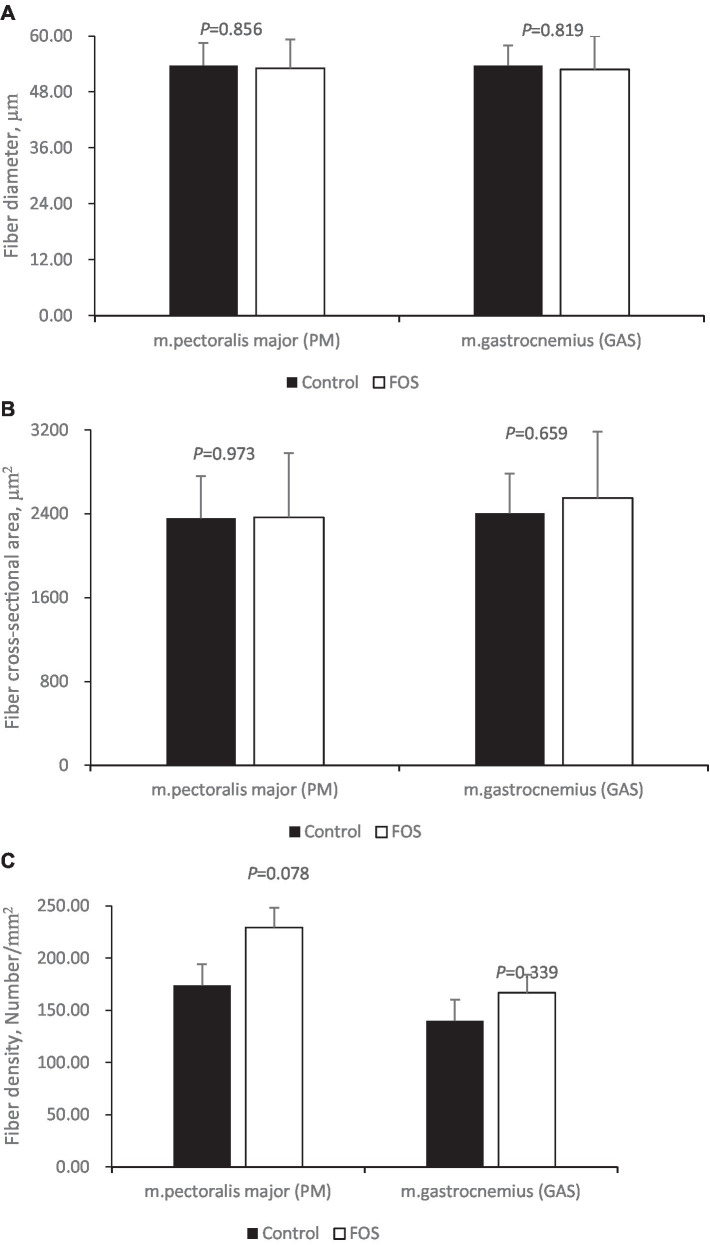
Muscle fiber morphology traits in breast (*pectoralis major*, PM) and thigh (*gastrocnemius*, GAS) muscle from control or 0.5% fructo-oligosaccharide (FOS) groups of Xianghuang broilers. Fiber diameter (**A**, μm), fiber cross-section area (**B**, μm^2^), fiber density (**C**, Number/mm^2^).

**Figure 2:**
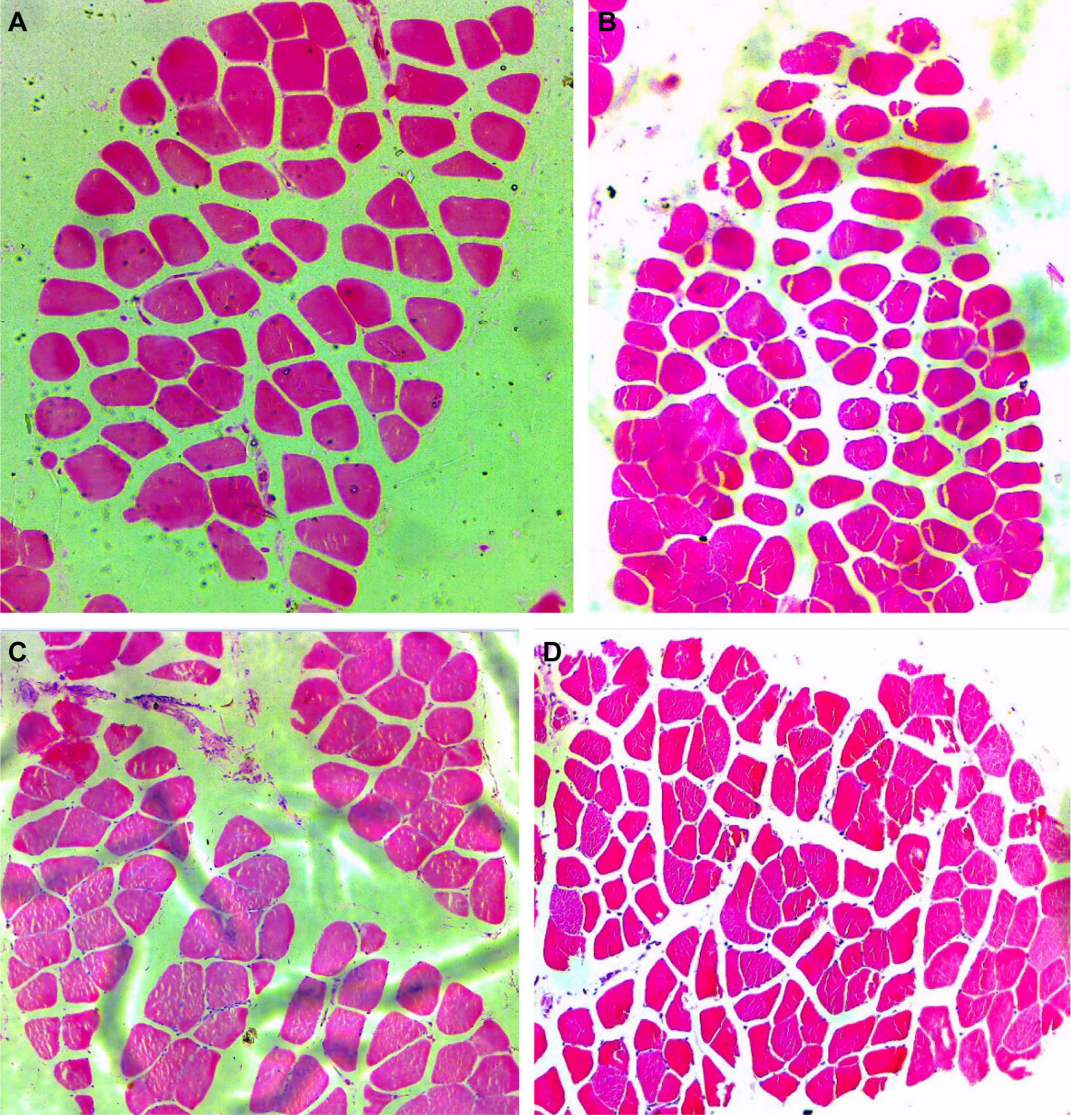
Hematoxylin and eosin staining in breast (*pectoralis major*, PM) and thigh (*gastrocnemius*, GAS) muscle from control or 0.5% fructo-oligosaccharide (FOS) groups of Xianghuang broilers. Magnification of 10*10 was used. PM in control group **(A)**, PM in FOS group **(B)**, GAS in control group **(C)**, GAS in FOS group **(D)**.

Dietary FOS supplementation did not affect fiber diameter (Figure 1A), cross-sectional area (Figure 1B) of breast (Figures 2A,B) or thigh muscle (Figures 2C,D) (*P* > 0.05). Muscle breast fiber density in FOS group tended to be higher than that in CON group (Figure 1C) (*P* = 0.078), but the fiber density of thigh muscle was the same between FOS and CON group (*P* > 0.05).

### 3.5 Textural parameters

**Figure 3:**
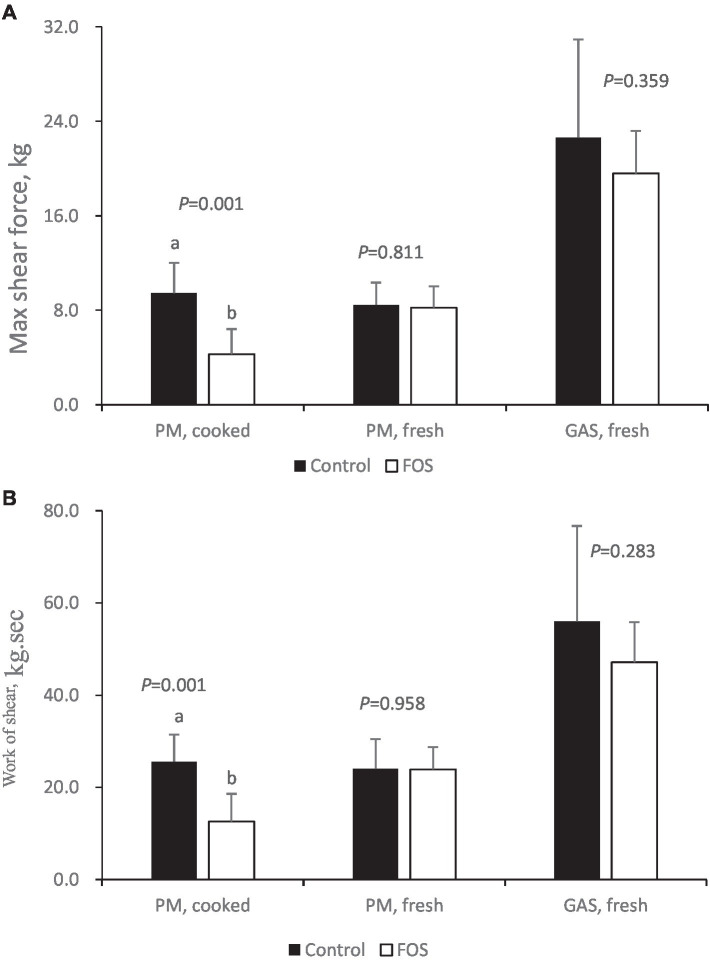
Toughness of cooked or fresh breast (*pectoralis major*, PM) and thigh (*gastrocnemius*, GAS) muscle from control or 0.5% fructo-oligosaccharide (FOS) groups of Xianghuang broilers. Probe: HDP/BSW. Max shear force **(A)**, Work of shear **(B)**. Data were presented as mean ± standard error of the mean. a, b Differs significantly at *P* < 0.05.

For toughness parameters, there were no significant differences between groups in regards to max shear force and work of shear of fresh breast (*pectoralis major*, PM) and thigh (*gastrocnemius*, GAS) muscle in broilers (*P* > 0.05) (Figure 3A). Max shear force and work of shear of cooked breast muscle was lower in FOS group compared with CON group (*P <* 0.05) (Figure 3B).

Fresh breast muscle in FOS group showed higher resilience compared to the CON group (Table 6). Adhesiveness of fresh thigh muscle tended to decrease (*P* = 0.080) in FOS group compared with CON group. Hardness (*P* = 0.065), fracturability (*P* = 0.063), gumminess (*P* = 0.079), chewiness (*P* = 0.080) of cooked thigh meat tended to be higher in FOS group compared to the CON group. Whereas, no significant differences were observed in the other TPA parameters such as adhesiveness, springiness, cohesiveness, resilience between the FOS and CON groups within fresh and cooked muscle (*P* > 0.05).

**Table 6:** Effect of fructo-oligosaccharides on texture properties of muscle in Xianghuang broilers.

Items^1^	Control	FOS	*P*-value
Breast muscle, fresh			
Hardness, g	193.82 ± 76.58	151.6 ± 71.11	0.306
Fracturability, g	151.95 ± 60.3	119.06 ± 55.75	0.310
Adhesiveness, g.sec	−11.55 ± 2.77	−13.93 ± 4.29	0.241
Springiness	0.98 ± 0.01	0.98 ± 0.01	0.943
Cohesiveness	0.56 ± 0.04	0.58 ± 0.03	0.263
Gumminess	107.86 ± 38.5	88.57 ± 40.57	0.379
Chewiness	105.52 ± 36.97	86.57 ± 39.03	0.370
Resilience	0.11 ± 0.06^B^	0.15 ± 0.08^A^	0.023
Thigh muscle, fresh			
Hardness, g	251.93 ± 134.91	242.12 ± 145.7	0.898
Fracturability, g	209.96 ± 115.07	201.21 ± 119.55	0.891
Adhesiveness, g.sec	−2.16 ± 1.11	−2.77 ± 1.43	0.080
Springiness	0.98 ± 0.00	1.00 ± 0.18	0.812
Cohesiveness	0.63 ± 0.03	0.65 ± 0.03	0.243
Gumminess	157.57 ± 85.2	154.45 ± 89.16	0.948
Chewiness	154.78 ± 83.58	152.21 ± 87.28	0.956
Resilience	0.33 ± 0.07	0.36 ± 0.13	0.539
Breast meat, cooked			
Hardness, g	1253.91 ± 255.51	1203.95 ± 364.11	0.771
Fracturability, g	1143.52 ± 235.03	1100.03 ± 339.71	0.785
Adhesiveness, g.sec	−0.24 ± 0.22	−0.30 ± 0.26	0.668
Springiness	1.2 ± 1.02	1.11 ± 0.66	0.855
Cohesiveness	0.71 ± 0.03	0.71 ± 0.03	0.748
Gumminess	895.38 ± 191.95	855.20 ± 277.33	0.758
Chewiness	1153.24 ± 1231.48	896.42 ± 372.41	0.607
Resilience	0.45 ± 0.29	0.35 ± 0.03	0.375
Thigh meat, cooked			
Hardness, g	466 ± 290.55	617 ± 321.22	0.065
Fracturability, g	416 ± 268.6	556 ± 292.78	0.063
Adhesiveness, g.sec	−0.24 ± 0.20	−0.43 ± 0.23	0.479
Springiness	1.44 ± 0.67	0.98 ± 0.29	0.772
Cohesiveness	0.65 ± 0.11	0.62 ± 0.18	0.423
Gumminess	303.58 ± 197.93	400.72 ± 215.74	0.079
Chewiness	303.37 ± 197.75	400.37 ± 215.6	0.080
Resilience	0.55 ± 0.42	0.41 ± 0.17	0.608

1 Values in a row without common superscripts differ significantly at *P* < 0.05.

### 3.6 Antioxidant function

The MDA concentration and T-SOD activity of breast muscle tended to be lower in FOS group compared with CON group (*P* = 0.066) (Table 7). Activities of T-AOC, CAT, GSHPX of breast muscle did not differ significantly from each other (*P* > 0.05). Broilers in FOS group showed lower T-SOD activity in thigh muscle compared with CON group (*P* < 0.05). There was an increasing trend for T-AOC activity of thigh muscle in FOS group compared to CON group (*P* = 0.053). There were no significant differences in concentration of MDA and activities of CAT, GSHPX of thigh muscle between FOS and CON groups (*P* > 0.05). Dietary FOS supplementation did not affect MDA concentration and activities of T-AOC, T-SOD, CAT in liver of broilers (*P* > 0.05).

**Table 7:** Effect of fructo-oligosaccharides on lipid peroxidation and antioxidant activity in Xianghuang broilers.

Items^1^	Control	FOS	*P-*value
Breast muscle			
MDA, mmol/g prot	107.89 ± 40.97	67.82 ± 32.81	0.066
T-AOC, mmol/g prot	0.42 ± 0.03	0.45 ± 0.05	0.172
T-SOD, U/mg prot	21.84 ± 3.16	19.2 ± 1.37	0.066
CAT, U/mg prot	5.64 ± 3.91	7.22 ± 5.33	0.540
GSHPX, U/g prot	83.62 ± 42.02	95.82 ± 13	0.477
Thigh muscle			
MDA, mmol/g prot	81.62 ± 37.48	73.48 ± 33.92	0.678
T-AOC, mmol/g prot	0.36 ± 0.03	0.39 ± 0.01	0.053
T-SOD, U/mg prot	23.17 ± 1.28^A^	17.39 ± 4.30^B^	0.005
CAT, U/mg prot	13.39 ± 11.09	13.3 ± 6.44	0.986
GSHPX, U/g prot	132.57 ± 71.32	103.52 ± 73.36	0.467
Liver			
MDA, mmol/g prot	21.59 ± 19.69	15.44 ± 11.18	0.486
T-AOC, mmol/g prot	0.30 ± 0.06	0.27 ± 0.02	0.165
T-SOD, U/mg prot	6.62 ± 3.22	6.83 ± 1.03	0.873
CAT, U/mg prot	30.54 ± 7.7	25.67 ± 6.16	0.215

1 MDA, malonaldehyde; T-AOC, total antioxidant capacity; T-SOD, total superoxide dismutase; CAT, catalase; GSHPX, glutathione peroxidase. Values in a row without common superscripts differ significantly at *P* < 0.05.

### 3.7 Gene expression

Expression of genes related to inflammation and antioxidant function in muscle and liver were shown in Table 8. Hepatic genes’ mRNA expression such as heme oxygenase 1 (HMOX1), nuclear factor, erythroid 2 like 2 (NFE2L), CAT, SOD1, NAD(P)H quinone dehydrogenase 1 (NQO1), thioredoxin reductase 1 (TXNRD), interleukin 1, beta (IL1β), interleukin 8-like 2 (IL8L2), tumor necrosis factor alpha (TNFa) were not affected by dietary FOS supplementation (*P* > 0.05). The mRNA expression of NFE2L, CAT, SOD1, NQO1,TXNRD were higher in FOS-fed broiler breast compared to the CON diet (*P <* 0.05). There was no significant difference in HMOX1, IL1β, IL8L2, TNFa mRNA expression in breast muscle between FOS and CON groups (*P* > 0.05). In thigh samples, expressions of HMOX1, TXNRD, SOD1 were down-regulated by FOS supplementation compared to the control group (*P <* 0.05). Birds from FOS group expressed higher IL1β in thigh muscle than that in CON group (*P <* 0.05). Gene expression of NFE2L, CAT, NQO1, and IL8L2 in thigh muscle were not affected by dietary FOS treatment (*P* > 0.05).

**Table 8:** Effect of fructo-oligosaccharides on gene relative mRNA expression in liver and muscle of Xianghuang broilers.

Items^1^	Control	FOS	*P*-value
Breast muscle			
HMOX1	1.34 ± 1.19	3.29 ± 2.65	0.093
NFE2L	2.03 ± 1.63^B^	7.39 ± 6.72^A^	0.049
CAT	2.97 ± 2.68 ^B^	8.89 ± 6.68 ^A^	0.034
SOD1	0.68 ± 0.55 ^B^	23.14 ± 18.32 ^A^	0.004
NQO1	1.50 ± 0.79 ^B^	5.64 ± 3.21 ^A^	0.008
TXNRD	0.76 ± 0.49 ^B^	14.22 ± 11.53 ^A^	0.006
ILIβ	1.05 ± 0.32	0.68 ± 0.6	0.131
IL8L2	2.10 ± 2.62	2.43 ± 2.97	0.820
TNFa	0.85 ± 0.52	1.76 ± 1.33	0.099
Thigh muscle			
HMOX1	1.34 ± 0.94^A^	0.32 ± 0.20 ^B^	0.010
NFE2L	3.37 ± 5.31	0.27 ± 0.19	0.148
CAT	3.26 ± 3.52	2.34 ± 0.95	0.516
SOD1	1.25 ± 0.67 ^A^	0.47 ± 0.26 ^B^	0.008
NQO1	1.08 ± 0.43	0.88 ± 0.91	0.603
TXNRD	1.23 ± 0.86 ^A^	0.25 ± 0.24 ^B^	0.012
ILIβ	1.59 ± 1.53 ^B^	4.03 ± 1.63 ^A^	0.008
IL8L2	1.60 ± 1.66	1.28 ± 0.69	0.646
TNFa	2.99 ± 3.65	8.02 ± 5.53	0.050
Liver			
HMOX1	1.86 ± 2.11	2.63 ± 2.92	0.524
NFE2L	1.03 ± 0.56	1.42 ± 0.93	0.312
CAT	10.72 ± 10.96	12.76 ± 12.14	0.714
SOD1	2.31 ± 2.95	0.63 ± 0.27	0.130
NQO1	1.45 ± 1.28	1.22 ± 0.79	0.661
TXNRD	3.24 ± 4.74	0.60 ± 0.30	0.137
ILIβ	0.80 ± 0.32	1.04 ± 0.49	0.246
IL8L2	2.36 ± 2.99	1.69 ± 1.35	0.564
TNFa	0.55 ± 0.58	0.67 ± 0.25	0.576

1 HMOX1, heme oxygenase 1; NFE2L, nuclear factor, erythroid 2 like 2; CAT, catalase; SOD1, superoxide dismutase 1; NQO1, NAD(P)H quinone dehydrogenase 1; TXNRD, thioredoxin reductase 1; IL1*β*, interleukin 1, beta; IL8L2, interleukin 8-like 2; TNFa, tumor necrosis factor alpha. Values in a row without common superscripts differ significantly at *P* < 0.05.

## 4 Discussion

Dietary FOS supplementation at 0.5% (5 g/kg) did not affect growth performance during late-growing period. This was the same that FOS did not affect ADG of broilers at 0.5% when compared with control group (25). Our earlier report showed that 200 mg/kg FOS had positive effect on ADG during first 5 weeks in chicken (26). No significant differences in breast, thigh yields were reported after dietary inclusion of 0.2 or 0.4% fructo-oligosaccharides (27). Earlier published studies also showed different results when considering of effects of FOS on growth performance of poultry. Birds given 0.6 g/kg fructo-oligosaccharides had lower ADFI and ADG compared with wheat based control group (28). Feeding 1.2 g/kg of inulin or 1.5 g/kg of FOS had a positive effect on ADFI and ADG of Archer Abro broilers aged 21 to 42 days (29). Study showed that trimmed asparagus by-products which contain 1.84% fructo-oligosaccharide led to higher ADFI, ADG at 30 and 50 g/kg but not 10 g/kg in Ross broiler chicks during first 0–25 days compared with control group (30). Synbiotic which containing probiotic and fructo-oligosaccharides showed an increasing effect on body weight of 42-day-old broilers subjected to daily cyclic heat stress episodes (31). Inulin which consists of fructose and glucose appeared to change the intestinal microbiota and showed a negative effect on growth performance before day 21 but positive effect subsequently up to day 42 (32). It seems that dosage of FOS and the age of broiler would affect the effect of FOS on growth performance.

In the present study, dietary inclusion of 0.5% FOS showed an increase in pH_45min_ value of breast muscle. If inclusion proportion was as low as 0.1 or 0.2%, FOS supplementation will not influence pH and water holding capacity (WHC) of chicken meat (27). Higher muscle pH could reflect slower speed of muscle glycogen degradation after slaughter (33). High pH in FOS could be a result by enhancing Bifidobacterium growth in small intestinal and cecal digesta which confirmed by early report (4.0 g/kg FOS) (11). Oxidative stress after slaughter could speeds up pH drop (34). A higher ultimate pH value in the breast or thigh muscle may be related to less oxidative stress. The decrease tendency in MDA accumulation of breast muscle indicated that lipid peroxidation of meat decreased in FOS group. Lipid, protein carbonyls, and endogenous reducing sugars may promote the initiation of Maillard reactions, and lead to formation of compounds, this oxidation reaction might reduce protein solubility and enhance denaturation and aggregation (35). pH value exhibited significant negative correlation with yellowness and Warner Bratzler shear force (36). Our result confirmed that broilers from FOS treatment showed lower toughness in breast muscle compared with control group.

Supplementation 0.5% FOS resulted in lower T-SOD activity and lower SOD1 gene expression of thigh muscle compared with control group in present study. The effect of FOS on antioxidant function could be different when dosage was not the same. Report showed that inclusion of 0.1 or 0.2% FOS in broilers’ diet showed no significant difference in free radical inhibition percentage expressed by ABTS (2,2 azino-bis-3-ethyl benzothiazoline-6-sulfonic acid) values and DPPH (2,2-diphenyl-1-picrylhydrazyl) values in fresh meat (27). But serum T-AOC increased and hepatic MDA reduced when FOS was at 0.3, 0.5, or 0.7% in broilers’ diet (25). Inulin could protect breast muscle by elevating SOD activity when birds challenged with *Clostridium perfringens* (37). Preventing myoglobin from being oxidized could improve the meat color. The b* value of thigh muscle was the same in FOS and CON. Though interleukin 1 beta gene expression increased and heme oxygenase 1, thioredoxin reductase 1 mRNA expression decreased, the muscle percentage and MDA concertation of thigh muscle were not affected by 0.5% FOS supplementation in this study. FOS (3.5 g of fiber/100 g of the mixture) could decrease the firmness of low-fat meatballs when compared with the control (38). A higher level of pH value in thigh muscle may indicate better tenderness, meat color and water holding capacity (39). But hardness of cooked meat tended to be higher in broilers from FOS group compared with control group. Breast muscle had lower fiber cross-sectional area and higher fiber density than those of thigh muscle (17). Fiber type composition can influence postnatal meat quality. The freezing storage conditions of test cooked meat samples prior to texture analysis might also contribute textural differences between treatments. And it seems that breast and thigh muscle respond differently to dietary FOS especially on firmness.

## 5 Conclusion

In conclusion, 0.5% of FOS supplementation did not affect growth performance of slower-growing Xianghuang broilers. Furthermore, FOS at 0.5% in diet might help to mitigate oxidate stress and then improve meat quality traits through increasing pH value, yellowness and tenderness of muscle.

## Data Availability

The datasets presented in this article are not readily available because no. Requests to access the datasets should be directed to Can Yang, yangcansky@163.com.
